# Soluble immune checkpoints reflect immune activation and treatment response in high-risk systemic sclerosis patients treated with plasma exchange

**DOI:** 10.1016/j.jtauto.2026.100361

**Published:** 2026-02-19

**Authors:** Judith Potjewijd, Rachid Tobal, Daan P.C. van Doorn, Leon J. Schurgers, Peter Heeringa, Jan G.M.C. Damoiseaux, Pieter van Paassen

**Affiliations:** a Department of Internal Medicine, Division Clinical and Experimental Immunology, Maastricht University Medical Center, Maastricht, the Netherlands; b Department of Biochemistry, Cardiovascular Research Institute Maastricht, Maastricht University, Maastricht, the Netherlands; c Department of Pathology and Medical Biology, University of Groningen, University Medical Center Groningen, Groningen, the Netherlands; d Central Diagnostic Laboratory, Maastricht University Medical Center, Maastricht, the Netherlands

**Keywords:** Systemic sclerosis, Soluble immune checkpoints, B cell activating factor, Cytokines, Matrix Gla protein, T cell exhaustion

## Abstract

**Introduction:**

Systemic sclerosis (SSc) is an autoimmune disease characterized by immune dysregulation, vasculopathy, and fibrosis. High-risk patients, particularly those with diffuse cutaneous involvement (dcSSc) and interstitial lung disease (ILD), may benefit from early intensive immunosuppressive therapy during the inflammatory phase of the disease. We hypothesized that soluble immune checkpoints (sICPs) reflect immune activation and serve as biomarkers for disease activity and treatment response.

**Methods:**

Plasma levels of 15 sICPs (including sPD-1, sTIM-3, sBTLA, sCD25, sCD137, sIDO), cytokines, B cell activating factor (BAFF) and dephosphorylated-uncarboxylated Matrix Gla Protein (dp-ucMGP) were analyzed in a prospective SSc cohort (n = 35). A high-risk subset (n = 14) received an intensified immunosuppressive regimen consisting of therapeutic plasma exchange (TPE), cyclophosphamide, and maintenance therapy with mycophenolate mofetil or rituximab in cases of intolerance. Samples were obtained at baseline, 6, and 12 months. Correlations with clinical variables and treatment response were assessed using non-parametric statistics and principal component analysis (PCA).

**Findings:**

Patients selected for intensified treatment had a distinct inflammatory profile with elevated levels of sICPs and BAFF, while CRP levels did not differ. sICPs correlated positively with dp-ucMGP, indicating a link between vascular dysfunction and immune activation. Classical pro-inflammatory cytokines (e.g., IL-6, TNFα), however, showed weak correlations with disease severity. Longitudinal analysis showed a significant decline in most sICPs within 6 months of treatment, whereas cytokine levels remained stable. Survival and pulmonary function were preserved during a median follow-up of 4.5 years.

**Interpretation:**

sICPs reflect T cell dysregulation and disease severity in SSc more accurately than classical cytokines. Early intervention in inflammatory, high-risk patients may prevent long-term clinical deterioration. The observed decline of sICPs following treatment supports their potential as early biomarkers of treatment response. Moreover, the correlation between sICPs and dp-ucMGP suggests a mechanistic link between vascular and immune pathology in SSc.

## 1 Introduction

Diffuse cutaneous systemic sclerosis (dcSSc) with interstitial lung disease (ILD) is an inflammatory, profibrotic phenotype associated with poor prognosis [1]. Consequently, treatment guidelines from the European Alliance of Associations for Rheumatology (EULAR) and the British Society for Rheumatology recommend autologous hematopoietic stem cell transplantation (AHSCT) in select cases [2,3]. AHSCT aims to reset the immune system by depleting autoreactive immune cells, potentially halting disease progression. Immune reconstitution has been observed and may contribute to immune tolerance [4]. However, because of high treatment-related toxicity and mortality, safer alternatives are needed. In a retrospective study, we demonstrated that intensified treatment with therapeutic plasma exchange (TPE) plus long-term immunosuppression offers comparable efficacy to AHSCT with a more favorable safety profile [5]. Other studies support intensified immunosuppressive treatments, such as rituximab (RTX) combined with mycophenolate mofetil (MMF), in severe disease [6]. These alternatives may benefit patients ineligible for AHSCT but still at risk of organ-threatening complications from cardiopulmonary involvement.

Early identification of patients with an inflammatory disease phenotype using reliable biomarkers is essential, as timely intervention may prevent organ damage and improve long-term outcomes. We previously identified elevated levels of dephosphorylated-uncarboxylated Matrix Gla Protein (dp-ucMGP) as a significant predictive risk factor for ILD development in SSc, also associated with increased mortality [7]. Matrix Gla Protein (MGP), a vitamin K–dependent inhibitor of vascular remodeling and calcification, helps maintain vascular homeostasis and regulates endothelial-to-mesenchymal transition (EndoMT), a process relevant to SSc vasculopathy and fibrosis [8]. The inactive form, dp-ucMGP, reflects functional vitamin K deficiency and impaired MGP activation. However, the specific role of dp-ucMGP in SSc-associated vasculopathy remains to be elucidated. In addition, immune checkpoints (ICPs) have emerged as key regulators of immune activation and self-tolerance. Through cell-cell interactions, ICPs modulate T cell activity, promoting or inhibiting immune responses. For example, programmed cell death protein 1 (PD-1) and its ligands (PD-L1, PD-L2) suppress T cell responses, whereas cytotoxic T lymphocyte-associated protein 4 (CTLA-4) inhibits T cell activation by competing with CD28 [9]. In autoimmune diseases, dysregulation of these pathways may cause inappropriate immune responses against self-antigens and tissue damage [10]. Beyond their membrane-bound forms, many ICPs also exist in soluble forms (sICPs), which circulate and may more accurately reflect systemic immune activity than cell-surface expression. Indeed, our recent work demonstrated that in a connective tissue disease cohort, plasma concentrations of multiple sICPs were significantly elevated compared with healthy controls, covering both co-stimulatory and co-inhibitory pathways [11].

In this study, SSc patients were selected for an intensified treatment approach including TPE based on clinical indicators of disease severity, reflecting presumed high inflammatory activity and immune dysregulation. These high-risk patients were prospectively followed for event-free survival (EFS) and pulmonary function. The primary objective was to identify immunological biomarkers predicting benefit and treatment response to intensified immunosuppressive strategies as alternatives to AHSCT. Plasma concentrations of cytokines and sICPs were measured and correlated with established clinical risk factors for severe disease. In addition, we explored the potential of dp-ucMGP as an inflammatory vascular biomarker in SSc. Longitudinal analyses assessed changes in cytokine and sICP levels over time under the intensified TPE-based protocol. This approach may support more precise immunological stratification of SSc patients, enabling personalized treatment strategies that improve safety and efficacy, especially for those not eligible for AHSCT.

## 2 Methods

### 2.1 Study design and participants

This single-center, observational, prospective cohort study included SSc patients enrolled at Maastricht University Medical Center, the Netherlands. The study adhered to the Declaration of Helsinki and Good Clinical Practice, with local ethics committee approval. Written informed consent was obtained from all participants. This study is registered at ClinicalTrials.gov (NCT03819777, 09-May-2018).

Adults with SSc classified according to current criteria [12] were included. Disease duration was defined as time from first non-Raynaud's manifestation. Use of oral corticosteroids (prednisone ≤7.5 mg daily) and hydroxychloroquine was permitted if patients were on a stable dose for ≥3 months before baseline; no other immunomodulatory therapies were allowed.

Patients meeting predefined criteria for severe disease were enrolled in our published protocol combining long-term immunosuppressive therapy with TPE [5]. Briefly, this intensified treatment included 7–10 sessions of TPE over 2–3 weeks in combination with 3-6 months of oral cyclophosphamide (2–3 mg/kg, adjusted for leukopenia), and maintenance therapy with either MMF or rituximab if intolerant. Eligible patients had disease duration <3 years (from first non-Raynaud symptom), age >18 years, and modified Rodnan skin score (mRSS) of ≥14 and/or pulmonary involvement or severe digital vasculitis. Pulmonary involvement and EFS were defined as in our previous work [5].

### 2.2 Collection and processing of samples

Blood plasma was collected at baseline and at 6 and 12 months after treatment initiation. Samples were drawn into EDTA tubes and centrifuged at 1300ˣg for 10 min at room temperature. Supernatant was extracted and stored at −80^o^C.

### 2.3 Measurement of dp-ucMGP

Circulating dp-ucMGP levels were measured in EDTA plasma in a single analytical run (Maastricht Laboratory of Coagulation Profile) using the InaKtif MGP chemiluminescent immunoassay (IDS-iSYS system, IDS, Boldon, UK) [13,14], as described previously [7,11]. Within-run precision was 0.8–6.2% and total precision 3.0–8.2%. Measurement range was 300–12,000 pmol/L, linear to 11,651 pmol/L.

### 2.4 Measurement of cytokines and chemokines by Luminex

Plasma cytokines and chemokines were quantified using a custom multiplex immunoassay with magnetic microspheres (Luminex, Austin, TX, USA) [15]. Capture antibodies were coupled to microspheres (50 μg/mL per 6.25 × 10^6 microspheres). Calibration curves were generated using recombinant proteins in serum diluent (Bio-Techne, Abingdon, UK). Positive and negative control microspheres were included as described by Skogstrand et al. [16]. Plasma (50 μL) was incubated with 10 μL microsphere suspension (500 microspheres/target) for 1 h at room temperature with shaking. After washing, 25 μL of secondary antibody cocktail (8 μg/mL per antibody) was added for 1 h. Streptavidin R-phycoerythrin (25 ng/well) was then added, followed by a final wash and resuspension in 100 μL HPE buffer. Data were acquired using the FlexMAP3D system (Bio-Rad) and analyzed with Bio-Plex Manager 6.1.1 (Bio-Rad). Assays were performed at the ISO 9001:2008-certified Multiplex Core Facility, Laboratory of Translational Immunology, University Medical Center Utrecht. Standards and assay variability are provided in Supplementary Table 1.

### *2.5* Measurement of BAFF

Plasma B-cell activating factor (BAFF) concentrations were measured using a commercial ELISA kit (Quantikine Human BAFF/BLyS/TNFΑSF13B, R&D Systems, Minneapolis, MN, USA) following the manufacturer's instructions. Plasma samples, diluted 1:2 with calibrator diluent, were added to anti-BAFF antibody-coated wells and incubated for 2 h at room temperature. After washing, horseradish peroxidase-conjugated anti-BAFF antibody was added for 2 h. Following another wash, substrate solution was added, developed for 30 min in the dark, stopped, and absorbance measured at 450 nm with 540 nm correction. BAFF concentrations were determined using a standard curve generated from recombinant BAFF standards. All samples were analyzed in duplicate.

### 2.6 Measurement of soluble immune checkpoints

Plasma concentrations of sICPs were quantified using a multiplex immunoassay (Life Technologies; ProcartaPL HU-IMM96T: EPX140-15803-901). Targets included B-and T-lymphocyte attenuator (BTLA), glucocorticoid-induced TNFΑR-related protein (GITR), herpesvirus entry mediator (HVEM), indoleamine 2,3-dioxygenase (IDO), lymphocyte activation gene 3 (LAG3), PD1, PD-L1, PD-L2, T-cell immunoglobulin and mucin domain 3 (TIM3), cluster of differentiation (CD)27, CD28, CD80, CD137 and CD152 (CTLA4/cytotoxic T-lymphocyte-associated antigen 4). Manufacturer protocols were followed. Patient samples were run in duplicates with 25 μL plasma each and measured using a Magpix (Luminex). Data were analyzed with xPONENT 4.2 software (Luminex). Values represent the mean of duplicates in picograms per milliliter. PD-L1 results fall outside the standard curve and were excluded.

### 2.7 Statistical analysis

Statistical analyses were performed using R version 4.4.1 (R Foundation for Statistical Computing, Vienna, Austria) in RStudio, SPSS Statistics for Windows version 28.0 (IBM, Armonk, NY), and GraphPad version 5.03 (Prism Software, San Diego, CA). Normally distributed variables are presented as mean ± standard deviation (SD) and skewed variables as medians (interquartile range [IQR]). Independent *t*-test, Mann-Whitney *U* test, and Chi square/Fisher's exact test were used as appropriate. One-way analysis of variance, Kruskal-Wallis *H*-test or Friedman test was used when >2 groups were compared. Pairwise correlations were computed using Spearman's rank coefficient.

Cytokines/chemokines, sICPs, sIL2R, CRP, dp-ucMGP, and BAFF concentrations were log10-transformed, scaled, and imputed for missing data using the “missMDA” R package. Principal component analysis (PCA) was conducted to reduce data dimensionality while preserving variance using R packages “FactoMineR” and “FactoInvestigate”. Dimensionality was evaluated from explained variance and visualized with scree plots. Group separation was tested using Kruskal–Wallis *H*-tests and post hoc Dunn's tests on the first two principal components (Dim1 and Dim2).

## 3 Results

### 3.1 General clinical characteristics

This study enrolled 35 SSc patients (mean age 58 ± 14.6 years), of whom 74% were female. ILD was present in 13 patients (37%) and PAH in 3 (8.6%). Fourteen patients met the predefined criteria for intensified treatment: 11 with ILD, 2 with diffuse cutaneous SSc, and 1 with severe digital vasculitis. All received TPE plus oral cyclophosphamide (median 3.5 months, IQR 3), followed by MMF (median 37.5 months, IQR 41) over a median follow-up of 45.6 months (IQR 27.6). Compared to the other patients, this subgroup had a shorter disease duration (0.6 vs. 3.8 years), more diffuse cutaneous disease (50% vs. 0%), higher mRSS (15.5 vs. 2), and more frequent pulmonary involvement (79% vs. 10%), in line with the predefined criteria for severe disease. Lung function at baseline did not differ significantly between groups (Table 1). Among the 21 patients who did not receive intensified therapy, 16 (76.2%) were deemed not to have an indication for immunosuppression. Of the remaining five patients, none fulfilled the predefined criteria for severe disease; however, three (14.3%) received MMF for mild skin or lung involvement, one (4.8%) methotrexate for arthritis, and one (4.8%) azathioprine combined with ciclosporin for digital vasculitis.

**Table 1:** Baseline clinical characteristics of SSc patients: Overall (n = 35), Intensified Treatment (n = 14), and Standard of Care (n = 21).

Clinical characteristics	SSc n = 35	Intensified treatment n = 14	SSc Standard care n = 21	p-value
**Age, y**	58.0 ± 14.6	59.7 ± 14.0	56.9 ± 15.2	0.556
**Female, n (%)**	26 (74)	8 (62)	18 (82)	0.112
**Disease duration^¥^, y**	1.1 (5.5)	0.6 (0.6)	3.8 (9.7)	**0.003**
**Disease ≤ 5 years, n (%)**	25 (71)	14 (100)	11 (52)	**0.002**
**Smoking, currently, n (%)**	4 (11)	1 (7)	3 (14)	0.809
**Skin involvement, n (%)**				**0.001**
Limited	23 (66)	5 (36)	18 (86)	
Diffuse	7 (20)	7 (50)	0 (0)	
Sine sclerosis	5 (14)	2 (14)	3 (14)	
**mRSS, score**	2 (11)	15.5 (24)	2 (2)	**0.001**
**Antibody, n (%)**				
Centromere	20 (57)	3 (21)	17 (81)	**0.006**
Topoisomerase I	8 (23)	5 (36)	3 (14)	0.221
RNA polymerase III	1 (3)	1 (7)	0 (0)	0.400
other∗	3 (9)	2 (14)	1 (5)	0.400
ANA only	2 (5)	2 (14)	0 (0)	0.153
None	1 (3)	1 (7)	0 (0)	0.400
**Digital ulcers, n (%)**	13 (37)	5 (36)	8 (38)	0.886
**ILD, n (%)**	13 (37)	11 (79)	2 (10)	**<0.001**
**Pulmonary hypertension, n (%)**	3 (8.6)	0 (0)	3 (14)	0.259
mPAP, mmHg, range	34 (33−50)			
PWP, mmHg, range	11 (10−12)			
PVR, dyn.sec.cm-5, range	571 (350−876)			
**FVC% predicted, %**	90 ± 17.7	86.3 ± 23.5	92.4 ± 12.7	0.065
**DLCO% predicted%**	68 ± 18.8	62.4 ± 18.7	72.6 ± 18.2	0.969
**Previous use of immunosuppressives, n (%)**	5 (14)			
Prednisone	1 (3)	0 (0)	1 (5)	1.000
Hydroxychloroquine	4 (11)	1 (7)	3 (14)	0.635
**Serum creatinine, μmol/L**	78.2 ± 21.5	78.1 ± 26.4	78.2 ± 18.2	0.282
**CRP, mg/L**	2 (3)	4 (15)	2 (3)	0.104

Count (percentage), mean ± standard deviation, and median (IQR) were used as appropriate. ANA: anti-nuclear antibodies; CRP: c-reactive protein; DLCO: diffusing capacity for carbon monoxide; FVC: forced vital capacity; ILD: interstitial lung disease; IQR: interquartile range; mPAP: mean pulmonary artery pressure; mRSS: modified Rodnan Skin Score; PVR: pulmonary vascular resistance; PWP: pulmonary arterial wedge pressure; SD: standard deviation. ¥ Defined as the date of the first non-Raynaud's phenomenon symptom. ∗Other: anti-Ku, Ro52, anti-*Pm*-ScL. Reference ranges for creatinine 60-115 μmol/L, CRP <10 mg/L.

### 3.2 Longitudinal follow-up: survival and pulmonary function

All 35 SSc patients were followed for a median of 53.5 months (IQR 40.5). Event-free survival at 72 months was similar between the intensified treatment group and the standard-of-care group (80.2% [standard error (SE) 10.5%] vs. 81.0% [SE 12.8%] at 72 months of follow-up; p = 0.706) (Figure not shown). No new ILD cases occurred in the intensified treatment group, versus one in the standard-of-care group; no new PAH cases were observed in either group. At the end of follow-up, mean FVC was 91.1% (± SD 27.2) in the intensified group and 93.5% (± SD 11.3) in the standard-of-care group, with ΔFVC of +4.7% vs. +0.2%, respectively (p = 0.077). Mean DLco was 68.1% (± SD 20.9) and 76.8% (± SD 22.7), respectively, with ΔDLco of +5.0% vs. +4.5% (p = 0.853).

### 3.3 Correlation of cytokines, sICPs, dp-ucMGP and clinical variables

**Fig. 1:**
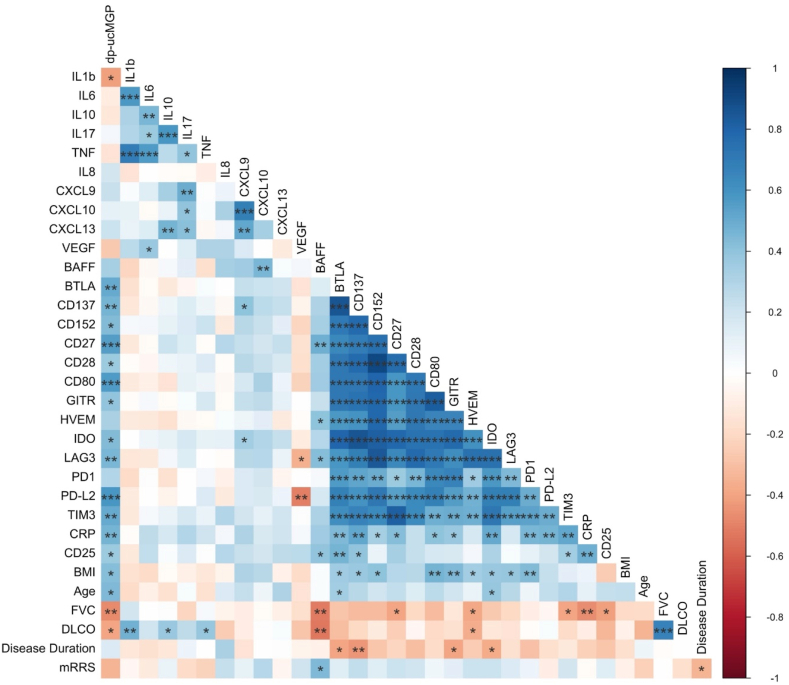
**Correlation matrix of cytokines, sICPs, dp-ucMGP and clinical variables in SSc patients**. Correlation matrix of baseline levels of cytokines, sICPs, dp-ucMGP and clinical variables in SSc patients (n = 35). BAFF: B cell activating factor; BMI: body mass index; BTLA: B- and T-lymphocyte attenuator; CD: cluster of differentiation; CRP: c-reactive protein; DLCO: diffusing capacity of the lung for carbon monoxide; dp-ucMGP: dephosphorylated-uncarboxylated Matrix Gla Protein; FVC: forced vital capacity; GITR: glucocorticoid-induced TNFR-related protein; HVEM: herpesvirus entry mediator; IQR: interquartile range; sICP: soluble immune checkpoint; IDO: indoleamine 2,3-dioxygenase; Lag-3: lymphocyte activation gene 3; mRSS: modified Rodnan Skin Score; PD-1: programmed cell death protein 1; PD-L2: programmed cell death-ligand 2; Tim-3: T-cell immunoglobulin and mucin domain 3; sCD25: soluble interleukin 2 receptor; VEGF: vascular endothelial growth factor.

An extensive pairwise correlation matrix was computed to investigate which immunological parameters best predicted clinical phenotype (see Fig. 1). Spearman's correlation coefficients were calculated between immunological and clinical variables, including mRSS, pulmonary function tests (FVC and DLco) and markers of inflammation**.** Classical inflammatory cytokines such as IL-6, IL-17, TNFα, CXCL9 and CXCL10 showed no strong correlation with mRSS or pulmonary function. In contrast, several sICPs (sHVEM, sCD27, sTIM3 and sCD25) negatively correlated with FVC and/or DLco. BAFF levels were positively associated with disease severity, as indicated by higher mRSS and worse FVC and DLco. Strong inter-correlations were observed among sICPs, while associations between sICPs and classical cytokines were limited, except for CRP and sCD25, which correlated broadly with sICPs. Dp-ucMGP levels showed positive correlations with most sICPs (except sPD1 and sHVEM), as well as with CRP and sCD25.

### 3.4 SSc patients selected for intensified treatment exhibit a more inflammatory profile

**Fig. 2:**
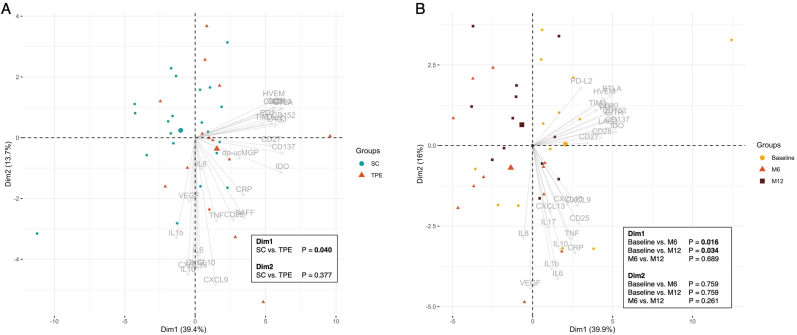
Biplot with Dim1 and Dim2of the baseline SSc cohort data (A), distinguishing between the intensified treatment group with therapeutic plasma exchange (red triangles) and standard care (green circles). Biplot of Dim1 and Dim2 of the longitudinal SSc cohort data (B). SC: standard care; TPE: therapeutic plasma exchange.

To compare SSc patients receiving intensified immunosuppressive treatment including TPE with those on standard care, a PCA was performed (Fig. 2A). The first two components (Dim1: 39.4%, Dim2: 13.7%) captured 53.1% of total variance. A significant separation along Dim1 (p = 0.040) indicated distinct inflammatory profiles between groups, whereas Dim2 showed no significant difference (p = 0.434).

Further analysis revealed that SSc patients selected for intensified immunosuppressive therapy, characterized by shorter disease duration, higher mRSS, and a higher prevalence of pulmonary involvement, had significantly elevated serum levels of sICPs, including sCD25, sBTLA, sCD137, sCD27, sGITR, sIDO, sPD-1, and sTim3, compared to the control group (Table 2). Additionally, increased concentrations of BAFF, the chemokines CXCL9 and CXCL10, and VEGF were observed in this group. In contrast, levels of CRP and dp-ucMGP did not differ significantly between groups.

**Table 2:** Baseline laboratory characteristics of SSc patients: Overall (n = 35), Intensified Treatment (n = 14), and Standard of Care (n = 21).

Laboratory characteristics	SSc n = 35	Intensified treatment n = 14	SSc Standard care n = 21	p-value
**CRP, mg/L**	2 (3)	4 (15)	2 (3)	0.104
**sCD25,U/mL**	454 (429)	743 (793)	360 (216)	**<0.001**
**dp-ucMGP, pmol/L**	566 (380)	597 (437)	509 (375)	0.426
**BAFF, ng/mL**	1.70 (1.1)	2.35 (3.7)	1.60 (1.0)	**0.032**
**sICP, pg/mL**				
sBTLA	1285.60 (1150.2)	1515.6 (1675.7)	983.9 (941.8)	**0.007**
sCD27	3654.30 (2983.1)	4431.9 (6721.7)	3100.0 (2764.1)	**0.048**
sCD80	178.40 (97.5)	197.9 (288.2)	143.3 (108.2)	0.235
sCD137	144.68 (90.7)	184.8 (125.1)	109.4 (73.1)	**0.002**
sCD152	44.2 (26.9)	51.8 (25.3)	41.1 (31.6)	0.263
sGITR	65.3 (41.2)	77.6 (44.6)	54.3 (46.8)	**0.044**
sIDO	84.10 (50.7)	96.4 (59.9)	68.5 (52.7)	**0.020**
sLag3	87.09 (75.8)	113.9 (81.4)	69.1 (71.2)	0.278
sPD-1	103.90 (79.4)	118.6 (72.4)	85.2 (84.7)	**0.048**
sPD-L2	2428.45 (1628.7)	2391.1 (1319.9)	2428.4 (2093.3)	0.829
sTim-3	2353.30 (1608.1)	2774.9 (1483.9)	2153.1 (1413.9)	**0.018**
**Cytokines, pg/mL**				
IL1b	0.006 (0.74)	0.001 (0.50)	0.19 (0.77)	0.181
IL6	7.21 (17.7)	6.81 (17.1)	7.61 (23.7)	0.576
IL8	18.8 (12.6)	22.2 (17.5)	16.9 (9.6)	0.169
IL10	2.09 (2.3)	1.68 (2.2)	2.17 (2.3)	0.624
IL17	4.42 (8.5)	3.60 (12.2)	4.50 (6.5)	0.807
TNFα	0.001 (0.74)	0.001 (0.37	0.007 (0.77)	0.148
CXCL9	193.8 (246.9)	269.8 (422.8)	169.9 (167.4)	**0.009**
CXCL10	429.3 (524.8)	833.2 (723.7)	367.4 (145.2)	**0.032**
CXCL13	50.2 (39.3)	54.8 (27.4)	43.6 (42.7)	0.344
VEGF	37.0 (38.5)	54.1 (33.1)	28.2 (29.8)	**0.027**

Medians with IQR are displayed. BAFF: B cell activating factor; BTLA: B- and T-lymphocyte attenuator; CD: cluster of differentiation; CRP: c-reactive protein; dp-ucMGP: dephosphorylated-uncarboxylated Matrix Gla Protein; GITR: glucocorticoid-induced TNFR-related protein; HVEM: herpesvirus entry mediator; IQR: interquartile range; sICP: soluble immune checkpoint; IDO: indoleamine 2,3-dioxygenase; Lag-3: lymphocyte activation gene 3; PD-1: programmed cell death protein 1; PD-L2: programmed cell death-ligand 2; Tim-3: T-cell immunoglobulin and mucin domain 3; sCD25: soluble interleukin 2 receptor; VEGF: vascular endothelial growth factor. Reference values for CRP <10 mg/L, sCD25 < 600 U/mL.

A separate PCA assessing inflammatory profiles across autoantibody subsets (anti-centromere, anti-topoisomerase, other) showed no distinct clustering (Suppl. Fig. 1).

### 3.5 Longitudinal assessment of sICPs and cytokine concentrations in SSc patients

To evaluate the impact of intensified immunosuppressive treatment, longitudinal samples were collected at baseline (M0), 6 months (M6), and 12 months (M12) from 9 SSc patients. PCA revealed that Dim1 and Dim2 accounted for 40.5% and 14.7% of the variance, respectively (total 55.2%) (Fig. 2B). Significant changes along Dim1 were observed between M0 and M6 (p = 0.009) and M0 and M12 (p = 0.031), indicating early immunological shifts that stabilized between M6 and M12 (p = 0.448). No significant differences were observed along Dim2.

**Fig. 3:**
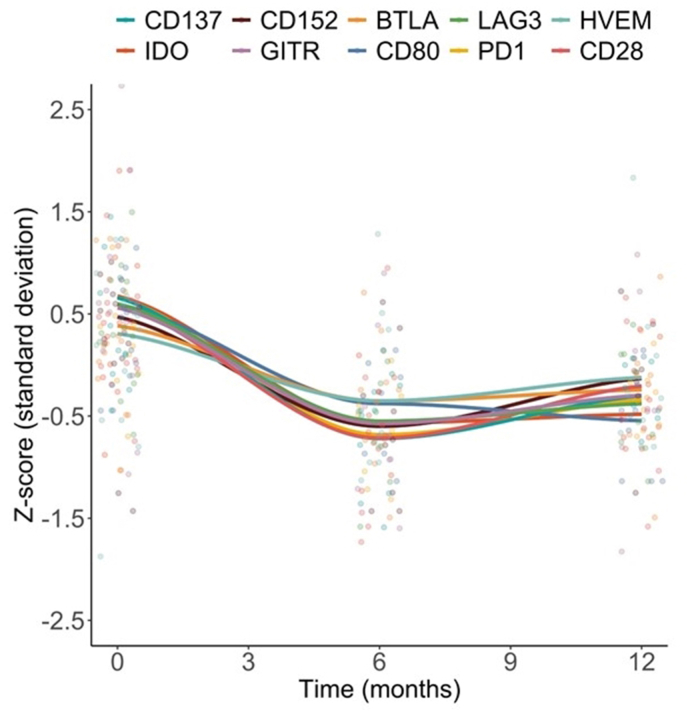
Z-scores over time during follow-up in patients from the intensified treatment group for soluble immune checkpoint proteins (sICPs) that contributed most to the variance over time (n = 9). BTLA: B- and T-lymphocyte attenuator; CD: cluster of differentiation; IDO: indoleamine 2,3-dioxygenase; PD-1: programmed cell death protein 1; Lag-3: lymphocyte activation gene 3.

Fig. 3 shows standardized Z-scores for the most responsive sICPs over time. Quantitative analysis confirmed significant declines in nearly all sICP levels at M6 and M12 (Table 3), while cytokine levels remained stable, suggesting that sICPs are more sensitive to treatment effects in this cohort.

**Table 3:** Longitudinal sICP and cytokine concentrations at baseline, 6 and 12 months after start intensified treatment, n = 9.

Laboratory characteristics	Baseline	6 months	12 months	p-value
**sCD25, U/mL**	734 (631)	398 (260)	394 (151)	**0.012**
**BAFF ng/mL**	2.35 (3.7)	2.00 (2.3)	2.50 (1.4)	0.180
**sICP, pg/mL**				
sBTLA	1358.7 (839.5)	889.8 (562.6)	952.8 (389.0)	**0.018**
sCD27	4440.0 (7552.1)	3474.9 (2000.9)	4076.3 (2296.3)	**0.008**
sCD28	329.0 (292.5)	114.3 (137.6)	141.3 (195.3)	**0.045**
sCD80	158.9 (208.5)	147.2 (91.7)	162.8 (91.7)	0.328
sCD137	144.7 (94.3)	101.1 (43.6)	103.9 (29.6)	**0.005**
sCD152	51.2 (28.8)	22.7 (22.6)	34.7 (14.7)	**0.027**
sGITR	68.0 (45.4)	48.8 (30.3)	43.3 (30.3)	**0.002**
sHVEM	432.5 (181.2)	247.9 (158.4)	228.6 (184.2)	**0.003**
sIDO	89.1 (47.8)	49.8 (25.8)	61.2 (19.1)	**0.001**
sLag3	119.2 (77.6)	50.2 (69.7)	69.1 (48.6)	0.159
sPD-1	103.9 (96.4)	57.6 (40.7)	87.0 (66.0)	**0.005**
sPD-L2	1842.1 (1778.4)	2337.7 (1140.5)	2838.2 (1035.0)	**0.013**
sTim-3	2569.5 (1733.3)	2098.6 (823.2)	2244.8 (950.5)	0.097
**Cytokines, pg/mL**				
IL1b	0.001 (0.49)	0.001 (12.07)	0.001 (0.89)	0.846
IL6	6.81 (17.12)	2.65 (15.87)	5.15 (14.33)	0.918
IL8	22.19 (17.48)	23.53 (18.04)	21.52 (15.91)	0.264
IL10	1.68 (2.22)	3.21 (9.06)	2.38 (1.60)	0.459
IL17	3.60 (12.19)	5.12 (14.39)	2.86 (9.57)	0.549
TNFα	0.001 (0.37)	0.001 (0.53)	0.001 (0.00)	0.307
CXCL9	296.83 (422.89)	209.01 (640.35)	174.24 (113.47	0.097
CXCL10	833.18 (723.74)	422.94 (1146.73)	429.11 (292.87)	0.121
CXCL13	54.80 (27.41)	90.15 (75.84)	39.37 (33.36)	0.264
VEGF	54.13 (33.09)	55.16 (45.48)	43.07 (31.56)	0.097

Medians with IQR are displayed. BAFF: B cell activating factor; BTLA: B- and T-lymphocyte attenuator; CD: cluster of differentiation; CRP: c-reactive protein; dp-ucMGP: dephosphorylated-uncarboxylated Matrix Gla Protein; GITR: glucocorticoid-induced TNFR-related protein; HVEM: herpesvirus entry mediator; IQR: interquartile range; sICP: soluble immune checkpoint; IDO: indoleamine 2,3-dioxygenase; Lag-3: lymphocyte activation gene 3; PD-1: programmed cell death protein 1; PD-L2: programmed cell death-ligand 2; Tim-3: T-cell immunoglobulin and mucin domain 3; sCD25: soluble interleukin 2 receptor; VEGF: vascular endothelial growth factor. Reference values for sCD25 < 600 U/mL.

## 4 Discussion

In this study, we identified a distinct immunological profile in high-risk SSc patients, supporting the rationale for stratified treatment approaches, including intensified immunosuppressive protocols. Our findings demonstrate that sICPs and BAFF more accurately reflect disease severity and immune dysregulation than classical pro-inflammatory cytokines, particularly in patients with ILD or dcSSc. The marked decline in sICP concentrations following intensified therapy, despite stable cytokine levels, underscores their potential as sensitive biomarkers of immunological response. Importantly, after a median follow-up of 4.5 years, survival and pulmonary function did not differ between the high-risk group receiving intensified treatment and the standard-of-care group, which predominantly exhibited limited cutaneous disease (lcSSc). This suggests that early intervention in high-risk patients may prevent long-term clinical deterioration. Moreover, our findings indicate that immunological profiling can aid in guiding treatment decisions, particularly in selecting patients who may benefit from intensified immunosuppressive therapy.

Recently, there has been increasing focus on identifying patients in the early inflammatory phase of SSc. Risk factors such as male sex, elevated CRP levels, anti-topoisomerase I antibody positivity, the diffuse cutaneous subtype, and short disease duration have been associated with more aggressive disease courses and poorer prognosis [17,18]. Consistent with this, patients selected for intensified therapy in our cohort had shorter disease duration, higher mRSS, and more pulmonary involvement. They also exhibited a distinct inflammatory profile characterized by elevated sICPs, BAFF, CXCL9, CXCL10, and VEGF. By contrast, CRP levels did not differ between groups, underscoring the limitations of conventional inflammatory markers and supporting the use of immunological profiling to identify patients at risk of progression. It is well recognized that only approximately 25% of patients with dcSSc have elevated CRP levels [19]; sICPs may therefore help fill this gap by identifying mechanism-based immune activation in high-risk patients, enabling earlier intervention to prevent progressive fibrotic organ damage.

To maintain self-tolerance and prevent autoimmunity, ICPs regulate immune responses via co-stimulatory and co-inhibitory pathways [20]. Soluble forms of these receptors and ligands (sICPs), generated through alternative mRNA splicing or proteolytic cleavage, circulate in plasma and modulate immunity by interacting with their membrane-bound counterparts. Their potential roles include competing for ligand or receptor binding, activating downstream signaling through direct binding, or integrating into membrane-bound ICPs to inhibit signal transduction [9,20]. Our findings demonstrate that multiple sICPs, including sPD-1, sTIM-3, sBTLA, sIDO, sCD25, sCD27, sCD137, and sGITR, are elevated in patients with early, severe SSc disease, and decrease markedly following immunosuppressive treatment.

Upregulation of co-inhibitory molecules, such as PD-1, TIM-3, and BTLA is a hallmark of T cell exhaustion, a dysfunctional state arising from chronic antigen exposure. In SSc, persistent immune stimulation may drive these pathways, contributing to disease progression. Elevated soluble forms have been associated with disease activity, severity, and diffuse cutaneous involvement [[21], [22], [23], [24]]. In vitro, sPD-1 interferes with PD-L1/2 signaling [25], while sTIM-3 blocks galectin-9-mediated inhibition of T cell activation [26]. Although the role of sBTLA in SSc has not been characterized, its elevation in our intensified-treatment group suggests relevance to early inflammatory disease. We also observed elevated levels of co-stimulatory sICPs, such as sCD137, sCD27, sCD25 and sGITR. sCD25 and sCD27 have previously been linked to diffuse cutaneous involvement and correlate with mRSS [27,28]. Notably, sBTLA inhibits T and B cell activation via HVEM, whereas TNFSF14/lymphocyte T-related inducible ligand that competes for glycoprotein D binding to herpes virus entry mediator on T cells (LIGHT), competing for the same receptor, enhances immune activation and promotes fibroblast profibrotic responses [29]. In SSc, both LIGHT and HVEM are upregulated in skin [30], and together with elevated sCD137 (TNFRSF9) and sGITR (TNFRSF18), these findings indicate a broader dysregulation of TNF-superfamily receptor–ligand interactions driving immune activation and fibrosis [31].

T cell exhaustion in SSc is characterized by impaired lymphocyte proliferation, diminished cytokine production, and persistent expression of inhibitory receptors. Our findings indicate that elevated sICPs may serve as peripheral markers of this dysfunctional state. Moreover, exhaustion may impair the regulatory function of both Tregs and regulatory B cells (Bregs), further exacerbating immune imbalance [32]. Supporting this, Fleury et al. demonstrated correlations between sICP levels and exhaustion markers in SSc, and showed that functional blockade of TIGIT and TIM-3 failed to restore cytokine production, suggesting irreversible exhaustion in a subset of patients [32]. Together, these data support a central role for altered ICP signaling in SSc pathogenesis and highlight sICPs as promising biomarkers of disease activity and treatment response.

In addition to T cell dysfunction, B cell abnormalities are a hallmark of SSc pathogenesis [33]. Altered B-cell homeostasis, including reduced memory B cell numbers and expansion of naïve B cells, is driven in part by elevated BAFF levels [34,35]. The BAFF/BAFF-R axis plays a critical role in B cell maturation, survival, and activation. Excess BAFF can rescue autoreactive B cells from deletion and promote the survival of inflammatory plasma cells. In vitro, BAFF enhances IgG and IL-6 production, promoting both autoantibody generation and pro-fibrotic signaling [33,34]. Previous studies have reported elevated BAFF levels in SSc, particularly in patients with skin fibrosis [35]. In line with these findings, our study demonstrates that BAFF levels correlate with both higher mRSS and reduced pulmonary function, supporting a role for B cell abnormalities in SSc pathogenesis, implicating it as a central player in SSc-related autoimmunity and tissue damage.

Our longitudinal analysis showed that most sICP levels declined significantly within 6 months of intensified immunosuppression and remained low at one year, indicating sustained suppression of immune cell activation. In contrast, cytokines remained stable, indicating that sICPs may be more sensitive indicators of treatment response. Importantly, sICPs were assessed 6 and 12 months after completion of the short-term PLEX cycle. The persistence of reduced sICP levels at these later time points, in the absence of concomitant changes in cytokine concentrations, argues against a transient treatment-related washout effect. Instead, this pattern suggests a more durable restoration of immune balance, indicating that sICP dynamics primarily reflect disease-related immunological processes rather than direct procedural effects. These results support the role of sICPs in early monitoring, especially in severe SSc. AHSCT is currently the most effective treatment for immune reconstitution in SSc, associated with increased TCR diversity, restoration of Tregs and Bregs, and durable disease control [4,36,37], however its risks limit broader use. Our previous study showed that TPE plus long-term immunosuppression yields comparable survival rates with better safety [5]. In the current study, intensified therapy led to similar outcomes in the high-risk group compared to patients with lcSSc, supporting its effectiveness.

Vascular dysfunction is a central feature of SSc, and pulmonary vascular remodeling is critical in the development of ILD and pulmonary hypertension. Dynamic cellular interactions at the fibrovascular interface, such as endothelial cell (EC) activation and EndoMT have been observed [38]. MGP has been shown to regulate EndoMT and maintain vascular integrity [8]. Its inactive form, dp-ucMGP, reflects functional vitamin K deficiency and impaired MGP activation, thereby linking vitamin K metabolism to vascular and fibrotic pathology in SSc. Our previous work identified dp-ucMGP as a predictor of mortality in SSc and a potential biomarker for ILD development [7]. In the present study, we extend these findings by showing that dp-ucMGP correlates positively with systemic inflammatory markers, including CRP and several sICPs, but not with classical inflammatory cytokines. Interestingly, dp-ucMGP levels did not differ between the intensified treatment group and the standard-of-care group, despite the former displaying a more pronounced inflammatory profile and higher prevalence of ILD. This suggests that dp-ucMGP primarily reflects vascular pathology rather than systemic inflammation, potentially by promoting EndoMT in an inflammatory and immune-dysregulated environment. If vitamin K supplementation can reduce dp-ucMGP and thereby limit vascular remodeling has to be studied. Although vasculopathic manifestations were evaluated, subgroup sizes were insufficient to allow robust stratified analyses, and the present study should therefore be considered exploratory in this respect.

### 4.1 Limitations

This study has several limitations. Firstly, the sample size, particularly for the subgroup undergoing intensified immunosuppressive therapy with longitudinal follow-up, was relatively small, which may limit the generalizability of the findings and statistical power to detect subtle differences in biomarker trajectories. Secondly**,** the decline in sICPs following treatment suggests a therapeutic effect, however the absence of a randomized control group limits the ability to distinguish treatment effects from natural disease fluctuation. Thirdly, although a broad panel of sICPs was measured, functional validation of these molecules and their cellular sources was beyond the scope of this study. Furthermore, sICP levels were assessed in peripheral blood, which may not fully reflect local immune activity within affected tissues such as the lung or skin. Taken together, our data position sICPs as candidate biomarkers of mechanism-based disease activity in severe SSc, while acknowledging that their clinical utility requires confirmation in larger, phenotypically stratified cohorts.

### 4.2 Conclusions

In conclusion, this study identified a distinct immunological profile in high-risk SSc patients, characterized by elevated sICPs and BAFF, rather than classical pro-inflammatory cytokines. The marked decline in sICPs following intensified immunosuppression suggests that these proteins may serve as sensitive biomarkers of immune activation and treatment response. The positive correlation between dp-ucMGP and both sICPs and CRP indicates a potential link between vascular dysfunction and immune dysregulation in SSc. Early recognition of this high-risk subset may rely not only on clinical severity but also on a characteristic immunological signature, supporting the rationale for timely and aggressive therapeutic intervention.

## CRediT authorship contribution statement

**Judith Potjewijd:** Writing – original draft, Visualization, Validation, Investigation, Formal analysis, Data curation, Conceptualization. **Rachid Tobal:** Writing – review & editing, Resources, Investigation. **Daan P.C. van Doorn:** Writing – review & editing, Visualization, Methodology. **Leon J. Schurgers:** Writing – review & editing, Resources, Conceptualization. **Peter Heeringa:** Writing – review & editing, Resources, Conceptualization. **Jan G.M.C. Damoiseaux:** Writing – review & editing, Supervision, Methodology, Conceptualization. **Pieter van Paassen:** Writing – review & editing, Supervision, Data curation, Conceptualization.

## Funding

This research did not receive any specific grant from funding agencies in the public, commercial, or not-for-profit sectors.

## Declaration of competing interest

The authors declare that no funds, grants, or other support were received during the preparation of this manuscript.

## Data Availability

Data will be made available on request.
