# Supplementary material for: Soluble immune checkpoints reflect immune activation and treatment response in high-risk systemic sclerosis patients treated with plasma exchange
Source: J Transl Autoimmun. 2026 Feb 19;12:100361. doi: 10.1016/j.jtauto.2026.100361 (PMC12955193; doi:10.1016/j.jtauto.2026.100361)
Supplement: Multimedia component 1 [file mmc1.docx]

**Supplementary table 1. Standards and assay variability of Multiplex Core Facility**

| **MARKER** | **BEAD** | **STND**  **HIGH** | **STND**  **LOW** | **LOD** | **LLOQ** | **ULOQ** | **INTRA ASSAY** | | **INTER ASSAY** | | **recovery** | **dilution** |
| --- | --- | --- | --- | --- | --- | --- | --- | --- | --- | --- | --- | --- |
|  |  | pg/mL | pg/mL | pg/mL | pg/mL | pg/mL | CV(%) | SD(%) | CV(%) | SD(%) | serum (%) | (n-fold) |
| **IL1B** | 012 | 5.000 | 1,2 | 2,2 | 7,3 | 3.812,5 | 3,4 | 2,9 | 3,5 | 2,5 | 100,8 | - |
| **IL6** | 028 | 10.000 | 2,4 | 2,4 | 7,9 | 10.370,3 | 4,4 | 2,7 | 11,0 | 4,6 | 100,0 | - |
| **IL8** | 057 | 10.000 | 2,4 | 7,1 | 23,8 | 9.095,1 | 5,8 | 3,6 | 13,7 | 6,8 | 101,7 | - |
| **IL10** | 037 | 10.000 | 2,4 | 0,9 | 3,0 | 9.760,4 | 6,0 | 4,1 | 6,8 | 3,7 | 99,6 | - |
| **IL17** | 043 | 5.000 | 1,2 | 8,8 | 29,4 | 5.019,6 | 5,1 | 5,1 | 7,8 | 5,6 | 99,6 | - |
| **TNFα** | 060 | 5.000 | 1,2 | 1,4 | 4,7 | 4.911,8 | 3,0 | 1,8 | 7,6 | 0,6 | 99,6 | - |
| **CXCL9** | 061 | 5.000 | 1,2 | 2,4 | 7,8 | 5.039,2 | 7,1 | 5,8 | 12,9 | 6,0 | 101,4 | - |
| **CXCL10** | 062 | 5.000 | 1,2 | 3,5 | 11,6 | 4.908,1 | 2,3 | 1,9 | 14,7 | 4,9 | 99,7 | - |
| **CXCL13** | 087 | 5.000 | 1,2 | 3,8 | 12,5 | 4.689,0 | 5,4 | 4,0 | 11,7 | 6,3 | 99,0 | - |
| **VEGF** | 069 | 10.000 | 2,4 | 1,5 | 5,1 | 9783,9 | 4,2 | 2,3 | 14,2 | 1,4 | 102,1 | - |

LLOQ: lower limit of quantitation; LOD: limit of detection; ULOQ: upper limit of quantitation.

**Supplementary figure 1.** **Supplementary Figure 1. Biplot of baseline SSc cohort (Dim1 vs. Dim2), highlighting autoantibody subsets: anti-topoisomerase (ATA; red triangles) and anti-centromere antibodies (ACA, yellow circles).**

**
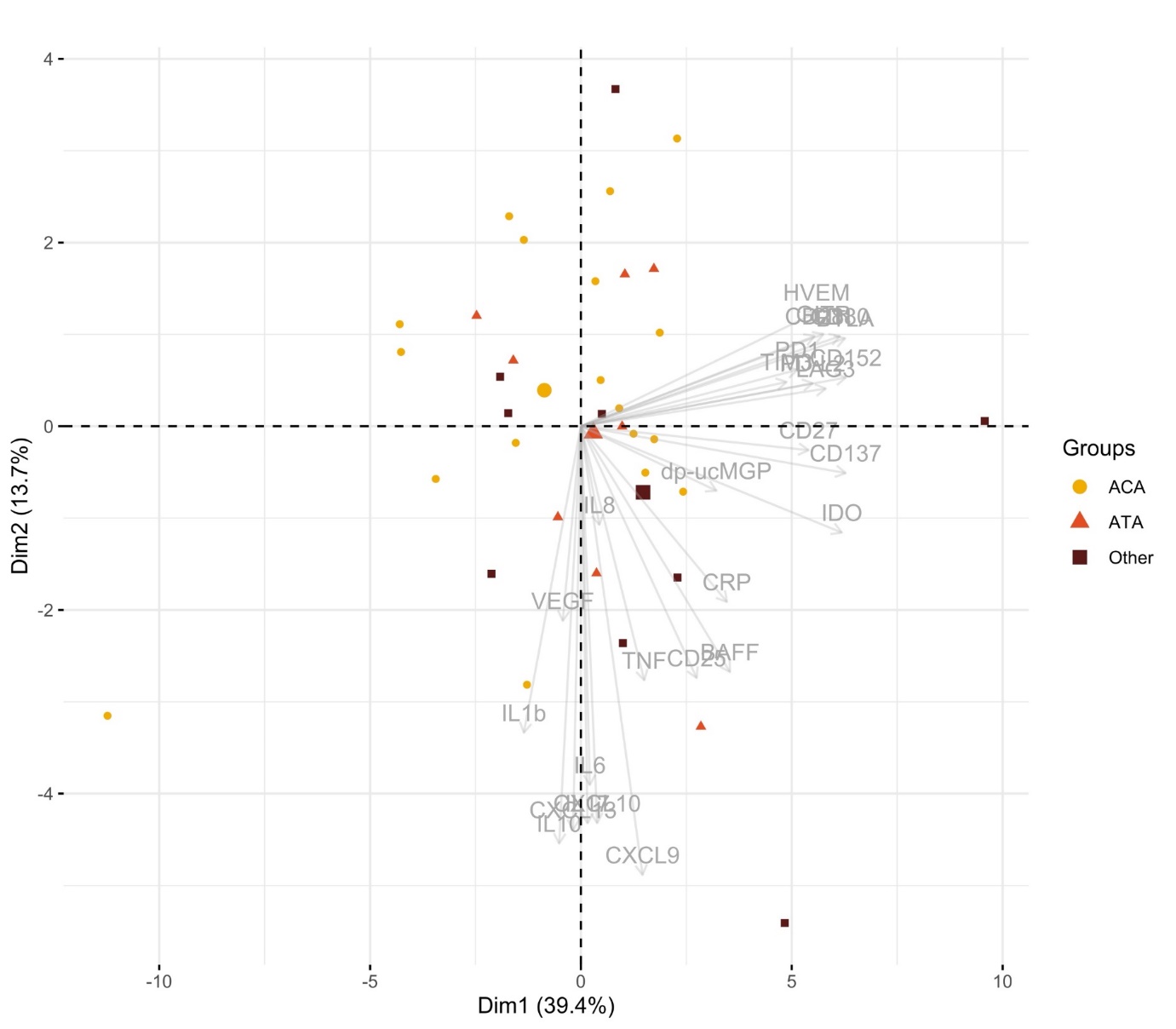
**

BAFF: B cell activating factor; BMI: body mass index; BTLA: B- and T-lymphocyte attenuator; CD: cluster of differentiation; CRP: c-reactive protein; DLCO: diffusing capacity of the lung for carbon monoxide; dp-ucMGP: dephosphorylated-uncarboxylated Matrix Gla Protein; FVC: forced vital capacity; GITR: glucocorticoid-induced TNFR-related protein; HVEM: herpesvirus entry mediator; IQR: interquartile range; sICP: soluble immune checkpoint; IDO: indoleamine 2,3-dioxygenase; Lag-3: lymphocyte activation gene 3; mRSS: modified Rodnan Skin Score; PD-1: programmed cell death protein 1; PD-L2: programmed cell death-ligand 2; Tim-3: T-cell immunoglobulin and mucin domain 3; sCD25: soluble interleukin 2 receptor; VEGF: vascular endothelial growth factor.
